# Copper – a scoping review for Nordic Nutrition Recommendations 2023

**DOI:** 10.29219/fnr.v67.10322

**Published:** 2023-11-27

**Authors:** Christine Henriksen, Erik Kristoffer Arnesen

**Affiliations:** Department of Nutrition, Institute of Basic Medical Sciences, University of Oslo, Oslo, Norway

**Keywords:** copper, trace elements, caeruloplasmin, nutrition recommendations

## Abstract

Copper functions as a structural component in many proteins involved in energy and iron metabolism, production of neurotransmitters, formation of connective tissue and endogenous antioxidant defence. Several biochemical indices have been suggested and used to assess copper status, but none of these has been found suitable for the detection of marginal copper deficiency or marginal copper toxicity. Copper imbalances have been linked to the pathogenesis of several chronic inflammatory diseases. During the last decade, a number of meta-analyses and systematic reviews have been published shedding light on the association between copper imbalances and some of these pathologies. Most of these meta-analyses are based on case–control studies. All show that blood copper concentrations are higher in cases than in controls, but there is inconclusive evidence to change the recommendations.

## Popular scientific summary

- Copper is an essential trace element for humans that is found in a variety of foods
- Copper is required for the functioning of several enzymes involved in the body’s energy and iron metabolism, production of neurotransmitters, formation of connective tissues and antioxidant defence
- No qualified, biomarker for copper status in the body is available
- Copper deficiency is rare in healthy humans
- Both low and high copper levels in the blood or tissues have been associated with chronic disease, such as Alzheimer’s dementia and cardiovascular disease, but the evidence is limited.

Copper (Cu) is an essential trace element with the atomic number of 29. It is a soft metal, with metallic-orange colour. Copper has two oxidation states (cuprous, Cu^+^ and cupric, Cu^2+^) and is involved in cell oxidation and reduction reactions. In the human body, copper functions as a structural component in a number of proteins as well as a catalytic cofactor of a number of enzymes involved in energy and iron metabolism, production of neurotransmitters, formation of connective tissues and endogenous antioxidant defence (1). Copper is found in a variety of foods, including grains, meat and nuts. Significant intakes may also come from tap water.

The aim of this scoping review is to describe the totality of evidence for the role of copper in health-related outcomes as a basis for setting and updating dietary reference values (DRVs) for the Nordic Nutrition Recommendations (NNR) 2023 (Box 1).

*Box 1.* Background papers for Nordic Nutrition Recommendations 2023

- This paper is one of many scoping reviews commissioned as part of the Nordic Nutrition Recommendations 2023 (NNR2023) project (2)
- The papers are included in the extended NNR2023 report, but, for transparency, these scoping reviews are also published in Food & Nutrition Research
- The scoping reviews have been peer reviewed by independent experts in the research field according to the standard procedures of the journal
- The scoping reviews have also been subjected to public consultations (see report to be published by the NNR2023 project)
- The NNR2023 committee has served as the editorial board.
- While these papers are a main fundament, the NNR2023 committee has the sole responsibility for setting dietary reference values in the NNR2023 project

## Methods

This scoping review follows the protocol developed within the NNR2023 project (2), and the sources of evidence used follow the eligibility criteria described by Christensen et al. (3). In the NNR2023 project, new scientific data and reasons for health concerns were first sought through a public hearing, which included scientists, authorities and the general public in the Nordic and Baltic countries. Existing systematic reviews (SRs) were identified primarily by contacting major national food and health authorities and organisations, and a general web search. Copper was not selected for a *de novo* SR for NNR2023, but one existing qualified SR on copper was identified by the NNR2023 project (4, 5).

A literature search was conducted in MEDLINE, with the following search string inserted into PubMed on 2023-01-17 using, (((copper[MeSH Terms]) AND ((‘2011’[Date – Entry] : ‘3000’[Date – Entry]))) AND (humans[Filter])) AND (copper[Title]), and applying the following filters: *Meta-Analysis, Systematic Review and English.*

This search identified 58 publications, published later than 2011. All were further scrutinised, and finally, 30 articles were judged as relevant and cited. Upon the publication of the NNR2023 report (2), an updated search was conducted by the NNR2023 committee on 15th April 2023, in which one additional publication was found to be eligible (6). Additionally, we have included three publications that were not picked up by the search string (1, 7, 8). Some references on copper physiology from NNR2012 (9) and a text book (10) are also included when relevant.

## Physiology

Copper is absorbed in the duodenum through active transport. At normal dietary intakes (1–5 mg/d), absorption varies between 12 and 60% and is regulated by the host status and dietary factors. The fractional absorption of copper decreases as the intake of copper increases. Absorption is inhibited by the presence of other minerals like zinc and iron, which can compete with copper for absorption. Additionally, certain compounds like phytates and oxalates bind to Cu^2+^ in the gastrointestinal tract and inhibit absorption (11). The relative absorption has been reported to be lower in lacto-ovo-vegetarian compared to non-vegetarian diets, but as the copper content was higher in the vegetarian diet, the net absorption was higher (12).

Dietary copper mainly exists in the Cu^2+^ redox state, and in order to be absorbed by the intestinal cell, Cu^2+^ needs to be reduced to Cu^+^ by a metalloreductase (13). Copper is then taken up by apical copper transporter 1 (CTR1). In the enterocytes of the small intestine, copper is either bound to a copper chaperone or is chelated by metallothionein, which is a protein that is induced by zinc and sequesters copper in the mucosal cells and prevents its transfer into the circulation (14, 15). Therefore, copper absorption is inhibited at high zinc intakes (>50 mg/d). The copper chaperones deliver the copper to the cellular-transporting proteins (e.g., ATP7A) for final secretion into circulation. In the circulation, copper is transported to the liver by albumin, transcuprein, low molecular weight copper histidine complexes, or a combination of these. Once absorbed into the liver, the main copper storage organ, copper is stored as metallothionein (16).

The release of copper from metallothionein makes copper available for other purposes, and it is transferred once again by the family of intracellular chaperones. Copper chaperones are small copper-binding proteins that deliver copper to specific copper-dependent proteins, and thereby both have a trafficking function and prevent copper from binding and damaging non-specific proteins (17).

For distribution to peripheral tissues, copper is incorporated into caeruloplasmin (70–90%) or albumin and transported by the circulation (1, 18, 19). Homeostasis of copper is regulated to some extent not only by absorption but also through excretion via bile, which can account for approximately 0.5–1.5 mg/d. Urinary excretion of copper is low.

The total body content of copper of an adult is approximately 50–120 mg, and 40% is found in bone and the rest in the liver and the brain (10). New-born infants have a larger amount of copper in the liver than adults, and this might act as a store of copper to be used for growth during the first couple of months.

There is substantial evidence from animal studies to suggest that diets low in copper reduce the activity of many of the copper-dependent metalloenzymes, e.g., cytochrome C oxidase, caeruloplasmin, dopamine hydroxylase, lysyl oxidase, superoxide dismutase involved in energy and iron metabolism, dopamine production, formation of elastin and collagen and the endogenous antioxidant defence. The activity of some of these metalloenzymes has also been shown to decrease during human copper depletion (20, 21).

## Assessment of nutrient status

Serum copper and whole blood caeruloplasmin concentrations are currently used as biochemical indices of copper status and can be used to detect severe copper deficiency. The decline in serum copper and caeruloplasmin concentrations observed when healthy young men were fed with a diet containing 0.38 mg/d of copper for 42 days was reversed by copper supplementation (22). In a number of other studies with higher levels of copper intake (0.66 mg/d and above), serum copper and caeruloplasmin concentrations did not decline significantly (23, 24), suggesting that this was a sufficient level of intake.

The dietary copper intake at which the serum caeruloplasmin concentration no longer increases in response to increased dietary copper might be considered the copper requirement. Other suggested indices of copper status include superoxide dismutase (SOD) activity, platelet copper concentration and cytochrome C oxidase activity because all of these have been shown to decline at low copper intakes. However, none of these indicators has been found suitable for the detection of marginal copper deficiency or marginal copper toxicity (11, 25). Instead, animal studies have suggested that one of the copper chaperones, CCS1, might be a potential biomarker for marginal copper deficiency and toxicity (26). At present, however, no robust, sensitive and specific biomarker for copper status is available (1, 27).

## Dietary intake in Nordic and Baltic countries

Copper is widely distributed in foods. Grains and meat products are the main sources in a Western diet (1). The highest levels of copper are found in liver, while milk and milk products have a low copper content. Nuts and seeds can also be a good source. Most grain products, meats, chocolate products, dried fruits, mushrooms, tomatoes, bananas and potatoes contain intermediate amounts. The intake of copper in the Nordic and Baltic countries varies between 1.1 and 2.1 mg/d (28). Dietary supplements usually contain copper in doses ranging from 0.4 to 1 mg.

## Health outcomes relevant for Nordic and Baltic countries

### Deficiency

Copper deficiency in humans is rare. Copper deficiency has been observed in formula-fed premature infants and cow’s milk-fed term infants recovering from malnutrition associated with chronic diarrhoea. It has also been reported in patients with prolonged total parenteral nutrition without additional copper and in patients after gastric bypass surgery (29). Symptoms of copper deficiency in children are low concentrations of white blood cells, anaemia, and hair and skin depigmentation (30). Heart and skeletal abnormalities have also been observed. Most of the symptoms can be related to deficiencies in copper-containing enzymes.

However, more recently, it has been argued that copper deficiency may have increased during the last couple of decades due to both hereditary and acquired factors. Examples of such factors would be changes in dietary habits and increased number of people undergoing bariatric surgery resulting in either decreased intake of copper or malabsorption (31).

One example of inherited copper deficiency is Menkes disease, also known as kinky hair disease. This rare condition is caused by mutations in the ATP7A gene, leading to a build-up of copper in certain parts of the body and a deficiency of copper in other parts, such as the hair and bones. Symptoms of Menkes disease include sparse, kinky hair, failure to thrive, developmental delays and seizures. Treatment typically involves copper supplements or in recent years preferably the copper-histidine complex (1, 32).

### Chronic diseases

Copper imbalances have been linked to the pathogenesis of several chronic inflammatory diseases, including metabolic disorders, cardiovascular disease (CVD), neurodegenerative diseases, endothelial dysfunction and skeletal muscle system dysfunction. During the last decade, a number of meta-analyses and SRs have been published shedding light on the association between copper imbalances and some of these pathologies. An overview of these publications is given in Table 1. Most of these meta-analyses are based on case–control studies only, and almost all show that blood copper concentrations are higher in cases than in controls (33–43).

**Table 1:** Meta-analyses and reviews, mostly based on case–control studies, of associations between copper status and health outcomes

Author	Methods	Outcome	Results
Chen et al. (44)	Meta-analysis	Abdominal aortic aneurysm	Aortic Cu levels are not different between patients and controls, but the Zn/Cu ratio is lower in patients.
Kim et al. (35)	Meta-analysis, 13 studies	Acute leukaemia	Serum Cu levels are positively associated with acute leukaemia
Squitti et al. (33)	Meta-analysis, 56 studies	Alzheimer’s disease	Brain Cu levels are decreased, and serum/plasma Cu levels are increased, in AD compared to controls. High serum/plasma Cu was associated with 3–4 times higher risk of AD.
Ventriglia et al. (45)	Meta-analysis, 15 studies	Alzheimer’s disease	Serum Cu levels are higher in AD compared to controls.
Loef and Walach (46)	SR of 101 studies, incl. SR, meta-analysis, RCT, observational, autopsy studies	Alzheimer’s disease	The SR suggests that a diet rich in Fe and Cu might aggravate the detrimental effect of a high intake of saturated fats on the risk of developing AD. Autopsy and case–control studies showed elevated levels of iron in brains from AD patients, while the evidence for Cu was less consistent.
Lewis et al. (31)	SR of six studies on Cu deficiency before and up to 12 months after bariatric surgery	Bariatric surgery	One study found an increase in deficiency after sleeve gastrectomy at 12 months compared to baseline; one study found an increased deficiency after RYGB at 6 months compared to baseline.
Kumar et al. (47)	SR of 22 case reports and one cohort study	Bariatric surgery with gastric bypass	Asymptomatic Cu deficiency after gastric bypass was found in ~10% of patients, while symptomatic deficiency is rare.
Mao and Huang (48)	SR meta-analysis of three studies	Bladder cancer	Serum Cu levels are increased in bladder cancer patients.
Wang et al. (41)	SR and meta-analysis of five RCTs	Blood lipids	Cu supplementation (2–8 mg/d) was not associated with change in total, LDL. or HDL cholesterol compared to control.
Rondanelli et al. (49)	Narrative review of 10 cross-sectional and RCTs	Bone metabolism	Due to the heterogeneity of the included studies, no conclusion can be made about the importance of copper in bone metabolism.
Feng et al. (38)	SR and meta-analysis of 36 studies on serum Cu and 16 studies on Cu/Zn ratio	Breast cancer	Serum Cu and Cu/Zn levels are higher in breast cancer patients compared to healthy controls or patients with benign breast diseases.
Jouybari et al. (50)	SR and meta-analysis	Breast cancer	Blood/serum, breast tissue, or hair levels of Cu were not significantly different for breast cancer patients compared to controls.
Zhang et al. (37)	Meta-analysis of 14 studies	Cervical cancer	Serum Cu levels were higher in cervical cancer patients compared to controls.
Ding and Zhang (8)	Meta-analysis of five studies on dietary Cu intake (case–control and cross-sectional)	Depression	Dietary Cu was inversely associated with depression (RR = 0.63)
Ni et al. (51)	SR and meta-analysis of 21 studies	Depressive disorder	Blood Cu levels were higher in patients with depression compared to controls. There was no difference in hair Cu levels.
Ressnerova et al. (52)	Review and meta-analysis	Head and neck cancer	Serum Cu levels were higher in patients compared to controls
Huang et al. (53)	Meta-analysis of 13 studies	Heart failure	Serum Cu levels were higher in patients compared to controls
Li et al. (54)	Meta-analysis	Hypertension	No significant difference in serum Cu levels between patients and controls.
Zhang et al. (39)	SR and meta-analysis of eight studies	Ischemic stroke	Ischemic stroke was associated with higher serum Cu levels compared to controls.
Zhang et al. (34)	SR and meta-analysis	Lung cancer	Serum Cu/Zn ratio was significantly higher in lung cancer patients compared to controls and benign lung disease patients.
Zhang and Yang (55)	Meta-analysis	Lung cancer	Serum Cu levels were higher in lung cancer patients compared to controls.
Ding et al. (7)	Meta-analysis of seven studies on dietary Cu (all cross-sectional)	Metabolic syndrome	Dietary Cu was inversely associated with metabolic syndrome (RR = 0.85)
Chen et al. (42)	Meta-analysis	Myocardial infarction	Serum Cu levels were higher in patients compared to controls.
Zheng et al. (56)	Meta-analysis	Osteoporosis	Serum Cu levels were lower in osteoporosis patients compared to controls.
Lin and Yang (36)	Meta-analysis	Ovarian cancer	Circulating Cu levels were higher in patients compared to controls. A Mendelian randomisation study did not find a significant association with risk of ovarian cancer.
Gu et al. (57)	Meta-analysis of 21 studies	Overweight/obesity	Serum Cu levels were not different between overweight and control groups but were higher in obesity.
Genoud et al. (58)	SR and meta-analysis of 18 studies	Parkinson’s disease	Serum/plasma or CSF levels were not different in patients compared to controls, but Cu in substantia nigra was significantly lower.
Adani et al. (59)	Meta-analysis of case–control studies of a total of 56 studies. For Cu, 10 studies on CSF and 28 studies on serum/plasma were included.	Parkinson’s disease	For Cu, CSF concentrations were (very) slightly higher and serum/plasma slightly lower in patients compared to controls
Jiang et al. (60)	Meta-analysis	PCOS	Women with PCOS had significantly higher serum Cu levels compared to controls.
Saghazadeh et al. (80)	SR and meta-analysis of 20 observational studies	Schizophrenia	Blood concentrations of Cu are higher in patients with schizophrenia than in mentally healthy controls. This is supported by studies done in patients with Wilson disease where mental disturbances have been observed before the onset of neurological and hepatic disorders
Shen et al. (61)	Meta-analysis	Thyroid cancer	Serum Cu levels were higher in patients with thyroid cancer compared to controls.
Qui et al. (43)	SR and meta-analysis of 15 observational studies	Type 1 and type 2 diabetes	Plasma and serum concentrations of Cu are higher in T1DM and T2DM, respectively, than in healthy controls
Eljazzar et al. (6)	SR of cross-sectional, cohort and interventional studies	Type 2 diabetes	Inconsistent associations (both positive and inverse) between Cu intake and risk of T2DM.

AD: Alzheimer’s dementia; CSF: cerebrospinal fluid; Cu: copper; HDL: high-density lipoprotein; LDL: low-density lipoprotein; PCOS: polycystic ovary syndrome; RYGB: Roux-en-Y Gastric Bypass; SR: systematic review; T1DM: type 1 diabetes mellitus; T2DM: type 2 diabetes mellitus; Zn: zinc.

Alzheimer’s dementia (AD) is an example of a health outcome where copper dyshomeostasis may be relevant. A meta-analysis of 56 studies investigating copper biomarkers in serum showed that serum copper excess was associated with a three to fourfold increase in the risk of having AD. Additionally, carriers of the *ATP7B* AG haplotype were significantly more frequent in the AD group. People with this genetic subtype seem, especially prone, to copper imbalance (33). A previous SR suggested that high intake of copper was associated with risk of AD in a setting of high intake of saturated fatty acids, but this was based on only one prospective cohort study (46). As studies assessing dietary copper intake and AD or cognitive decline are limited and unclear, European Food Safety Authority (EFSA) recently characterised the evidence for a causal relationship as ‘speculative and inconclusive’ (62).

Furthermore, several cancer types have been linked to copper imbalance in case–control studies. One meta-analysis of 39 papers showed that an elevated serum copper/zinc ratio might be associated with increased risk of lung cancer (34). The same pattern emerged in meta-analyses of 21 case–control studies of patients with leukaemia (35), 20 studies of patients with ovarian cancer (36), 14 studies in cervix caser (37), as well as 36 studies of women with breast cancer (38).

One major limitation with all these studies is the case–control or cross-sectional design, making it difficult to draw any conclusion about causality. Most circulating copper is bound to caeruloplasmin, and caeruloplasmin is an acute phase protein, which increases with inflammation. Therefore, one interpretation would be that this is the reason for the higher blood concentrations in the patients compared to controls. Only three of the SRs addressed the dietary copper intake. Therefore, the results cannot be interpreted in the way that increased intake of copper is linked to increase disease risk. Consequently, they cannot be used to set DRVs for copper.

CVDs have also been linked to high blood levels of copper, and meta-analyses have found increased serum copper in patients with myocardial infarction and ischaemic stroke compared with controls (39, 42). In contrast, a Mendelian randomisation study suggests that higher levels of genetically predicted copper might play a protective in the development of high blood pressure and coronary artery disease (40). Interestingly, the possibly protective effect of copper does not seem to be mediated through altered lipoprotein metabolism, but systolic blood pressure may be a mediator (41). On the other hand, there was no significant difference in serum copper levels in patients with hypertension compared to controls in a meta-analysis of cross-sectional and case–control studies (54). In conclusion, the evidence-linking copper status to CVD is non-consistent and should be further investigated.

### Toxicity

The intake of high doses of copper leads to acute toxicity, which includes symptoms of gastric pain, nausea, vomiting and diarrhoea. Storage of food in non-galvanised copper containers is associated with an increased risk of childhood cirrhosis in susceptible children (63). In areas with soft water, copper can leach from copper pipes and result in high copper concentrations (more than 100 mg/L) in drinking water, and gastro-intestinal disorders have been seen with intakes of copper-contaminated water containing 3.7 mg/L in infants (64). Infants are probably the most sensitive group, and case reports have indicated an association between high copper intake from water and symptoms of copper toxicity. Controlled and population-based studies found weak evidence for copper toxicity from drinking water at concentrations up to 2 mg/L (65), but local authorities may still recommend letting tap water run before it is used for consumption by infants, especially when used for formula.

The EU Scientific Committee on Food (SCF) proposed that an upper limit (UL) of 5 mg/d is safe for adults (66). This was based on the absence of negative effects on liver function during copper supplementation at 10 mg/day in one study and included an uncertainty factor of 2.0 to allow for potential variability within the normal population. In addition, the SCF noted that the UL was not applicable during pregnancy and lactation due to inadequate data. The U.S. Institute of Medicine (IOM, present National Academies of Sciences, Engineering, and Medicine; NASEM) set an UL for copper of 10 mg/d in 2001, largely based on the same data, but used an uncertainty factor of 1.0 (67). In 2023, the scientific committee of the EFSA published a reassessment of the acceptable daily intake (ADI) levels of copper, based on probability for retention in liver (62). The committee set an ADI of 0.07 mg/kg, which corresponds to 5 mg/day for a 70 kg person. This was well above the mean and 95th percentile of total dietary exposure to copper in adolescents, adults and elderly but below exposure in infants (62). However, due to higher expected copper requirements for growth, the intake observed in infants was considered unlikely to exceed the ADI throughout childhood.

## Requirements and recommended intake

### Adults

The precise requirement for copper is not known. Indications of deficient copper status, using SOD activity as a marker of copper status, have been reported with intakes of 0.7–1 mg/d (68–70). However, other studies in young men have not found indications of changes in copper status based on SOD activity, caeruloplasmin production, or plasma copper concentrations at intakes of 0.79 mg/d for 42 days (23). In a subsequent study in young men, an intake of 0.66 mg/d for 24 days followed by an intake of 0.38 mg/d for 42 days resulted in decreasing indicators of copper status over time (22, 71). Although the levels did not fall into the deficient range, a steady state was not completely reached. Other studies have shown that intakes below 0.7 mg/d are associated with increases in biomarkers related to disease, such as faecal-free radical production, faecal water alkaline phosphatase activity and cytotoxicity (72) or deceased immune function (73). There are, therefore, limited data to establish an average copper requirement for adults, but the available data suggest that an intake of approximately 0.7–0.8 mg/d will maintain adequate copper status based on plasma copper concentrations, caeruloplasmin production and SOD activity. The IOM based its recommended copper intake for adults on a number of indicators, including plasma and platelet copper concentration, serum caeruloplasmin concentration and erythrocyte SOD activity in controlled depletion-repletion studies (67).

In a report from EFSA (1), the panel considered that none of the biomarkers of copper status was sufficiently robust, sensitive and specific to be used to set requirements for copper. Instead, they based the DRV on balance studies, observed dietary intake data in the general population and proposed an adequate intake (AI) of 1.6 mg/day for men and 1.3 mg/day for women. In these studies (23, 74–77), a relatively small number of male and female volunteers (*n* = 8–21) were provided diets containing low, medium or high amounts of copper. The dietary periods varied from 10 to 120 days. Intakes below 1.29 mg copper/day resulted in all cases in negative copper balances calculated as difference between dietary intake and faecal excretion. Intake of either 1.29 mg copper/day (77) or 1.6 mg copper/day (74, 76) resulted in balances of 0.015 ± 0.44 mg, 0.06 mg and 0.00 ± 0.31 mg copper/day, respectively. However, only the study by Milne et al. (77) measured sweat and dermal losses only in 3 men. The sweat and dermal losses for these three men were on average 0.12–0.15 mg/day. If urine, sweat and dermal losses were included or adjusted for in the calculations, intake below 1.6 mg Cu/day would result in negative balances in males.

From the studies described earlier, it can be concluded that only intakes above 2.49 mg/d resulted in positive balances for males (74, 77, 78). However, these studies are small and imprecise. No new balance studies have been published since the NNR2012 edition.

### Children

The copper content of human breast milk is highest during early lactation and then declines over the course of lactation. The mean copper content of human breast milk during the first 6 months of lactation is approximately 0.25 mg/L (79), and there are no indications of inadequate copper status in breast-fed infants. For infants 6–11 months old and children ≥1 year old, the requirements are based on extrapolation from adults with allowance for growth.

### Pregnancy and lactation

The requirement for extra copper during pregnancy is relatively low. Based on the accumulation of copper in the foetus and maternal tissue during pregnancy, an additional 100 µg per day was suggested by the IOM. The copper content of human breast milk is approximately 0.25 mg/L. With a milk production of approximately 750 mL/d and an estimated copper absorption of 50%, an extra 0.3 mg/d of copper during lactation is recommended by the IOM.

## Conflict of interest and funding

The authors have not received any funding or benefits from industry or elsewhere to conduct this study.
